# Composition and biological significance of the human N^α^-terminal acetyltransferases

**DOI:** 10.1186/1753-6561-3-S6-S3

**Published:** 2009-08-04

**Authors:** Kristian K Starheim, Darina Gromyko, Rolf Velde, Jan Erik Varhaug, Thomas Arnesen

**Affiliations:** 1 Department of Molecular Biology, University of Bergen, N-5020 Bergen, Norway; 2 Department of Surgical Sciences, University of Bergen, N-5020 Bergen, Norway; 3 Department of Surgery, Haukeland University Hospital, N-5021 Bergen, Norway

## Abstract

Protein N^α^-terminal acetylation is one of the most common protein modifications in eukaryotic cells, occurring on approximately 80% of soluble human proteins. An increasing number of studies links N^α^-terminal acetylation to cell differentiation, cell cycle, cell survival, and cancer. Thus, N^α^-terminal acetylation is an essential modification for normal cell function in humans. Still, little is known about the functional role of N^α^-terminal acetylation. Recently, the three major human N-acetyltransferase complexes, hNatA, hNatB and hNatC, were identified and characterized. We here summarize the identified N-terminal acetyltransferase complexes in humans, and we review the biological studies on N^α^-terminal acetylation in humans and other higher eukaryotes.

## Background

The importance of N^α^-terminal acetylation in human cell biology and disease has been increasingly recognized. During the last five years, the major human N^α^-acetyltransferase complexes (NAT) have been identified and characterized. In humans as in yeast, three NAT complexes are believed to perform most N^α^-acetylations, namely the human NatA, NatB and NatC complexes (hNatA, hNatB, and hNatC) [1-4]. In addition, a potential hNatE complex has been described [5-7]. A number of studies have described various aspects of N^α^-terminal acetylation in humans, such as substrates, NAT knockdown phenotypes, and expression patterns of NAT subunits. Through these studies, a complex and specific system of N^α^-terminal acetylation has been revealed. This system is to a large extent conserved from yeast. Still, little is known about the function and regulation of the system, and of the specific mechanisms through which phenotypes are mediated. In this review we give a comprehensive overview of the knowledge of co-translational N^α^-terminal acetylation in humans and other higher eukaryotes.

## The human NatA complex

The NatA complex is the most thoroughly studied of the three major NAT complexes in higher eukaryotes. The NatA complex is believed to be the major NAT complex both in humans and in yeast: the number of potential hNatA substrates is high as compared to human NatB and human NatC (hNatB and hNatC). Also, the phenotypes resulting from hNatA knockdown appears to be slightly more severe than those observed for hNatB and hNatC knockdown [3,4,8].

### Composition of the hNatA complex

**Figure 1:**
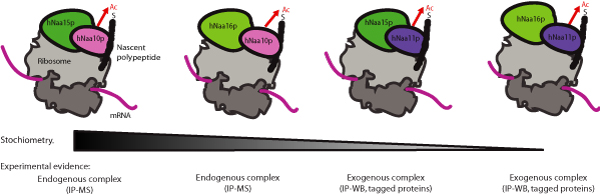
**Composition of the four different hNatA complexes**. The hNatA subunits hNaa10p, hNaa11p, hNaa15p and hNaa16p can combine to form four variants of the hNatA complex. All subunits tested bind to ribosomes (hNaa11p not tested yet), suggesting that all four variants can acetylate nascent polypeptides (e.g polypeptide with an N-terminal Serine) co-translationally. The gradient illustrates the expected abundance of the various complexes. Based on EST data and immunoprecipitation experiments [21], hNaa10p-hNaa15p forms the most abundant version of the complex, displaying a stochiometric relationship of 6:1 compared to the hNaa10p-hNaa16p complex in HEK293 cells. The hNaa11p-hNaa15p and hNaa11p-hNaa16p complexes are probably present to an even lesser extent in most tissues, except for tissues like testis, where hNaa11p is upregulated. In the lower part of the figure it is indicated which experimental data that forms the evidence of the complex formations. IP, immunoprecipitation; MS, Mass Spectrometry; WB, Western Blotting.

The hNatA complex is conserved from yeast with respect to subunit homology [1] and substrate specificity [9]. The most characterized human hNatA complex consists of the catalytic subunit hNaa10p (hArd1), and the auxiliary subunit hNaa15p (NATH/hNat1) [1,2,10]. They are orthologues of the yeast NatA components yNaa10p and yNaa15p. Both hNaa10p and hNaa15p are associated with ribosomes, suggesting a model where hNatA performs co-translational acetylation of nascent polypeptides [1]. Interestingly, a significant portion of hNaa10p and hNaa15p is also found to be non-ribosomal. Paralogues of hNaa10p, hNaa11p (hArd2), and of hNaa15p, hNaa16p (hNat2), have been suggested to participate in functional hNatA complexes [11,12]. This allows for four possible hNatA complexes, resulting in a more complex subunit composition in humans as compared to yeast (Figure 1). Based on expression sequence tag data (EST) from UniGene Cluster, and experimental evidence [12], we here describe hNaa10p and hNaa15p as components of the abundant form of the hNatA complex, and hNat11p and hNat16p as alternative and less abundant subunits in the hNatA complex (Figure 1).

#### hNaa10p

hNaa10p (hArd1) is a 235 amino acid protein with a theoretical mass of 25.4 kDa. It contains a conserved core motif responsible for acetyl coenzyme A binding (Q/RxxGxG/A), as found in all members of the GNAT superfamily of N-acetyltransferases (GNAT, Pfam: PF00583 Acetyltransf_1). hNaa10p is homologous to the yeast NatA catalytic subunit yNaa10p. It localizes to both the cytoplasm and the nucleus, and it is present both in a ribosome-bound and a non-ribosome-bound form [1]. The C-terminal part of hNaa10p is unstructured [13] containing several potential phosphorylation sites. Indeed, hNaa10p is phosphorylated on several of these sites [14,15]. At least some of the phosphorylations are mediated through the GSK-3 kinase [16]. This may point toward regulation of hNatA activity through phosphorylation of hNaa10p.

Mammalian Naa10p has several splice variants that are of biological interest. Studies on *M. musculus *have identified an evolutionarily conserved NatA complex, consisting of mNaa10p (mArd1) and mNaa15p (mNat1) [17]. Three splice variants were identified for mNaa10p (mArd1): mNaa10p^198^, mNaa10p^225^, and mNaa10p^235^, where mNaa10p^235 ^was considered as the wildtype [18]. mNaa10p^225 ^and mNaa10p^235 ^displayed differences in subcellular localizations, suggesting that they may differ in activity and function [19]. While mNaa10p^235 ^is a component of the mNatA complex together with mNaa15p, mNaa10p^225 ^was shown to N^ε^-acetylate a lysine residue of transcription factor hypoxia-inducible factor 1α (HIF-1α), and thereby destabilize the protein [20]. The mammalian Naa10p variants mNaa10p^235 ^and hNaa10p^235 ^did not destabilize HIF-1α [18]. In humans, only one isoform of hNaa10p have been characterized: the wildtype hNaa10p^235^, which is orthologous to mNaa10p^235 ^[19].

EST data show that h*NAA10 *(ref. no. Hs. 433291) is ubiquitously expressed in most tissues. Northern blot analysis of multiple human tissues showed h*NAA10 *expression in all studied tissues, with higher expression levels in brain, heart, liver, and skeletal muscle [20]. Several groups demonstrated hNaa10p expression at protein level in a broad range of human cancer cell lines, and also in human tissues [21,22].

#### hNaa11p

hNaa11p (hArd2) display 81% sequence identity to hNaa10p, and h*NAA11 *is the result of a mammal-specific retrotransposition event, making h*NAA11 *a gene duplicate of h*NAA10*. Exogenous hNaa11p displays N^α^-acetyltransferase activity and forms putative hNatA complexes in association with hNaa15p and hNaa16p [11,12]. The h*NAA11 *mRNA is moderately expressed in most tissues, and its function is largely unknown. In NB4 cells it was found that levels of hNaa10p and hNaa15p decreased during retinoic acid induced differention, while the level of hNaa11p remained stable [11], thus some difference in function may be expected between the proteins.

EST data show that h*NAA11 *expression is restricted to certain tissues (liver, placenta, skin, testis). Study of h*NAA11 *expression in human cell lines indicated expression in different human epithelial cells and promyelocytic leukemia cells. These levels were significantly lower than those observed for h*NAA10 *[11]. Recently, Pang and colleagues reported that the mouse orthologue of h*NAA11*, mouse *NAA11 *(m*NAA11*, m*ARD2*) was upregulated in testis during male meiosis [23]. It was not found upregulated in other somatic tissues, except for trace amount in the ovary. Interestingly, the testis developmental expression pattern of mNaa11p clearly indicated delayed translation of m*NAA11 *during spermatogenesis. This may be explained by a tissue specific role of mNaa11p at a later stage of the spermatogenic process, and a regulated role of m*NAA11 *different from that of m*NAA10*. As m*NAA10 *is located on the X-chromosome, the authors speculated that the increased m*NAA11 *expression is to compensate for the loss of m*NAA10 *expression during meiosis.

#### hNaa15p

The auxiliary subunit hNaa15p (NATH, hNat1) is a protein with a theoretical mass of 101.3 kDa. It is localized to the cytoplasm, where it interacts with both cytosolic and, in particular, cytoskeleton-bound polysomes [1]. Also, a large fraction of hNaa10p and hNaa15p are not ribosome-associated. This may indicate that the subunits can have roles other than those in a NatA complex. hNaa15p expression levels are positively correlated with hNaa10p expression levels *in vivo*. [24]. Observations in yeast [24] and in human cell culture [8,25] could point to hNaa15p positively affecting the level of hNaa10p.

h*NAA15 *is expressed in most adult human tissues at a low level. Various studies have shown that the expression of h*NAA15 *is correlated with high proliferation. Increased expression have been detected in highly proliferative tissues and cell lines such as Burkitt lymphoma cell line, colorectal carcinoma SW480 cell lines, testis, ovary, spleen, colon and stomach [10,26]. However, exogenous overexpression of h*NAA15 *in NPA and HEK293 cell lines did not alter cellular proliferation *per se *[10].

A series of studies has focused on the role of the mouse *NAA15 *splice variant *Tubedown-1 *(*Tbdn-1*) in development and differentiation, and the expression of mNaa15 (Narg1, Tbdb100) in neuronal development. As the sequence similarity beween human and mouse Naa10p and Naa15p is very high (99.2% and 99.7%, respectively), one might expect that results from mouse studies are highly relevant also for human systems. *Tbdn-1 *encodes a protein of 593 amino acids. This is considerably shorter than the 866 amino acids of hNaa15p. Both *Tbdn-1 *and m*NAA10 *were identified as embryonic genes that were expressed *in vivo *at relatively high levels in neural precursors, and downregulated during neuronal development.

The same tendency was found for m*NAA15 in vitro *in the mouse embryonic carcinoma P19 [17] and in mouse embryonic cell line (IEM) [27] when differentiation was induced. High expression of m*NAA15 *and m*NAA10 *remains in postnatal period at the sites of neurogenesis and synaptic plasticity like hippocampus and cerebellar cortex [17]. These findings suggest that a high expressional rate of mNatA may be a marker for immature cells being able to divide, or to undergo long term changes in formation of synapses. Also, Ohkawa and colleagues studied m*NAA15 *and m*NAA10 *expression postnatally in the cerebellum of developing neurons. They found increasing expression levels of m*NAA15 *and m*NAA10 *during Purkinje cell development. This could be an indication that N^α^-acetyltransferase activity of mNatA is linked to processes such as dendrogenesis and dendritic arborisation [28].

Endothelial-specific conditional knockdown of m*NAA15 *in bitransgenic mice led to neovascular rethinopathy [29]. These data are in accordance with findings that m*NAA15 *expression is suppressed both during oxygen-induced retinopathy in mice and during retinopathy of prematurity in humans [30], and in neovascular retinopathy associated with diabetes [27]. This may indicate that maintenance of m*NAA15 *is important both for retinal blood vessel homeostasis, and for preventing retinal neovascularization in adults.

Northern blot analysis of m*NAA15 *clearly demonstrated different distribution of gene expression in tissues and during development. In adult tissues m*NAA15 *level was relatively low, with exception of the atrial endocardium, the endothelial and myeloid regions of bone marrow, and in vascular bed of ovarian follicles [27]. These data indicate that m*NAA15 *may be involved in regulation of vascular and hematopoietic development, and physiological angiogenesis. Knockdown of m*NAA15 *in endothelial cells led to significant increase in cellular permeability, and knockdown *in vivo *in mice resulted in retinal neovascularization with formation of abnormal blood vessels prone to albumin leakage. Since mNaa15p was shown to interact with cortactin, a known regulator of cellular permeability, the observed m*NAA15 *RNAi phenotype was suggested to be due to impaired interactions between cortactin and mNaa15p. mNaa15p was also proposed to be a cortactin-associated controller of the retinal endothelial cells permeability to albumin [25].

The increased expression of m*NAA15 *and m*NAA10 *that was observed during postnatal dendrogenesis could be due to an essential role for N^α^-acetyltransferase activity in normal dendritic development. Interestingly, knocking down rat *NAA10*, or overexpressing the dominant negative forms of *NAA10 *dramatically limited dendrogenesis in cultured rat embryonic neurons. These data indicate that NatA positively regulates the growth and branching of dendritic extensions, this being necessary for retaining the plasticity of synapsis formation [28].

When differentiation of human neuroblastoma cells was induced by retinoic acid treatment *in vitro*, a significant decrease in hNaa15p expression was observed. This is in agreement with pattern of hNaa15p expression in neuroblastic tumors [31].

When differentiation of human NB4 promyelocytic leukemia cells was induced by retinoic acid, endogenous hNaa10p and hNaa15p was downregulated, while the hNaa11p level remained unchanged [11]. This differentiation pattern is in accordance with recently reported data from studies on mouse testis, and it is a strong argument for separate regulatory mechanisms of hNaa10p and hNaa11p during differentiation [23].

hNaa15p (Tbdn100) was also proposed to be an essential part of a Ku-transcriptional complex along with Ku70 and Ku80 [32]. This complex regulates osteocalcin gene expression in human osteosarcoma cells. Chromatin immunoprecipitation experiments confirmed that both Ku and hNaa15p were associated with the Osteocalcin promoter. This study implicated hNaa15 in a nuclear function, perhaps independent of hNaa10p and N^α^-terminal acetylation.

#### hNaa16p

An orthologue of hNaa15p, hNaa16p (hNat2) was recently described. It displays 70% sequence identity to hNaa15p, and the h*NAA16 *gene originates from an early vertebrate duplication event from the common ancestor of h*NAA15 *and h*NAA16*. As of today, the specific function of hNaa16p remains unknown [12]

Endogenous hNaa16p is found to interact with hNaa10p, and an exogenous complex between these two proteins is functional as an hNatA enzyme complex. Also, hNaa16p is found in both a ribosome-bound form and a non-ribosomal form in human cells, thus it may be a component of a co-translational hNatA complex.

EST data show that h*NAA16 *(ref. no. Hs. 512914) is expressed in a variety of human cell lines, but generally at lower levels as compared to h*NAA15 *(ref. no. Hs. 715706). h*NAA16 *expression have not been correlated with cell proliferation, as was observed for h*NAA15 *[12]. When comparing the expression of h*NAA15 *and h*NAA16*, h*NAA15 *seems to be the dominant variant in most tissues and cell types. For example, in HEK293 cells h*NAA15 *and h*NAA16 *are expressed in a 5:1 ratio at the mRNA level, and the resulting proteins are detected in complex with hNaa10p in a 6:1 ratio, respectively. Exceptions from this are tissues like adrenal gland, mammary gland, heart, testis, and thymus, where h*NAA16 *appears to be the dominant variant [12]. Expression patterns in mouse tissues also demonstrated that m*NAA15 *is the dominating species as compared to m*NAA16 *[17].

### hNatA acetylates a wide range of substrates involved in important cellular functions

The human NatA complex (hNatA) is coupled to removal of the initial methionine by Methionine aminopeptidase 1 and 2. Once the methionine is removed, the resulting N-terminus is available for acetylation by hNatA. The hNatA complex potentially acetylates Ser-, Ala-, Thr-, Gly-, Val- and Cys- N-termini. A recent proteomics study identified a large number of hNatA substrates, and the *in vivo *substrate specificity of hNatA was found to closely resemble that of yNatA [9]. The COFRADIC methology used in this study is presented elsewhere in this supplement (Van Damme *et al*.). This study detected proteins displaying significantly altered N^α^-acetylation after downregulation of both hNaa10p and hNaa15p in human cell culture [9]. The list of proteins was surprisingly short: only 17 proteins among several hundred acetylated hNatA type substrates analyzed showed a significant change in acetylation status. Most likely, the temporary knockdown of hNatA only partially affected some of the substrates, while the bulk of substrates were unaffected. Among affected substrates were proteins participating in protein-protein interactions (Ankyrin repeat domain-containin protein 44, WD-repeat protein 5), transcriptional regulation (Transcriptor elongation factor B, GCN1-like protein 1, density regulated protein), ribosome assembly (ribosomal protein L13a), protein folding (Cyclophilin A), RNA maturation (U6 snRNA-assosiated Sm-like protein LSm8), and protein modification (SAPK substrate protein 1, Ubiquitin-like 1-activating enzyme E1A). We hypothesize that Nα-acetylation may be important for substrate function, and that a decrease in N^α^-acetylation of some of these or undetected substrate proteins may induce the observed phenotypes associated with hNatA knockdown. Obviously, several important hNatA substrates are likely to be present in the pool of proteins unaffected by hNatA knockdown, but since complete removal of hNatA appears to be equivalent to cell death (see separate section below), these will be hard to detect by this methodology. Based on the substrate specificity and the N-terminal sequences of all known human proteins (SwissProt v. 56.0), it was estimated that more than 8000 unique human proteins (out of a total of around 20,000 unique proteins) are hNatA substrates [9].

There are some examples of possible downstream NatA substrates or effectors where a direct link has not been established, but where the biological importance makes it worth mentioning. These include hemoglobin subunits γ-globin and ζ-globin, β-amyolid precursor protein (APP), α-tubulin, β-catenin and HIF-1α.

The hemoglobin subunits γ-globin and ζ-globin are potential hNatA substrates due to their N-terminal sequences matching the hNatA substrate specificity. N^α^-terminal acetylation of these proteins leads to decreased O_2_-binding [33,34].

Through the interaction of hNaa10p with APP, hNatA may be linked to Alzheimers disease. The hNatA complex hNaa10p-hNaa15p could suppress the secretion of a processed form of APP, amyloid β-protein (Aβ) in mouse neuroblastoma cell line. Aβ is a main component of the pathological state that brings about Alzheimers disease. The finding that secretion of Aβ could be modulated by hNatA is interesting, but the mechanism is completely unknown. Although APP is a direct interactor of hNaa10p, its N-terminal sequence (MLPG) is unsuited as an hNatA substrate. Thus, it is still unclear if hNatA modulates Aβ secretion direct or indirect through N^α^-acetylation of one or more components of the Aβ secretion pathway, or if the effect is mediated through the direct interaction with APP [2].

NatA has been linked to dendritic growth in developing neurons. α-tubulin was proposed to be an hNatA substrate, and the effect on dendritic growth was shown to be mediated through the acetylation of α-tubulin. The N-terminus of α-tubulin (MRECI) does not match the hNaa10p substrate specificity, and the authors suggested that hNatA N^ε^-acetylates α-tubulin post-translationally [28]. This may indicate that Naa10p could act both as a NAT with N^α^-acetyltransferase activity, and as a HAT (Histone Acetyltransferase) with N^ε^-acetyltransferase activity. However, there is no evidence for this at present.

Indeed, hNaa10p has been suggested to act as an N^ε^-acetyltransferase also in other studies. Recently, hNaa10p was shown to acetylate and activate β-catenin in lung cancer cells, thereby promoting cell proliferation [35]. β-catenin has become an attractive target for studies due to its multiple roles in cancerogenesis, development and regeneration. It is an integral component of the Wnt signalling pathway, and in conjunction with T-cell factor 4 (TCF4) it functions as a transcriptor factor complex for many genes.

Another field of interest, with respect to the possible N^ε^-acetylation activity of hNaa10p, is the interaction between hNaa10p and hypoxia-inducible factor 1α (HIF-1α). hNaa10p (hNaa10p^235^) stably interacts with HIF-1α [36], but hNaa10p does not acetylate HIF-1α [22,36-38]. By binding with hNaa10p, HIF-1α inhibits hNaa10p-mediated activation of β-catenin/TCF4 complex, leading to repression of β-catenin transcriptional activity [39]. Thereby, a new mechanism was proposed, where an hNaa10p-HIF-1α interaction inhibits Wnt signaling, leading to hypoxia-induced growth arrest in tumors. Gene expression analysis of h*NAA10 *RNAi-treated HepG2 cells with hypoxia-treated samples showed overlap between h*NAA10*-regulated genes and hypoxia-regulated genes [37], strengthening the hypothesis that hNaa10p and HIF-1α may work in concert. More detailed information regarding this has previously been presented [40].

As we know of, no convincing proof has been given as to whether hNaa10p can perform N^ε^-acetylation *in vivo*. Future studies are needed to reveal whether hNaa10p in fact have a dual function as both a NAT and a HAT type of enzyme, or whether hNaa10p indirectly mediate N^ε^-acetylation.

### Knockdown studies point to a role for hNatA in cell cycle and apoptosis

**Figure 2:**
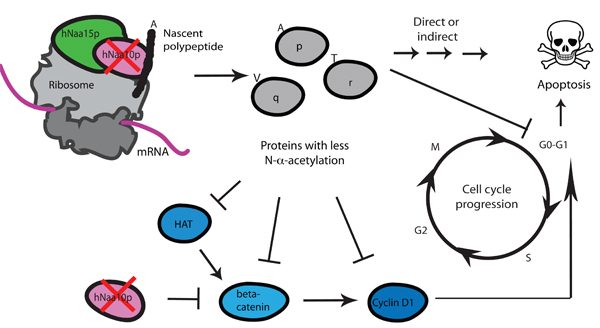
**Knockdown of h*NAA10 *of the hNatA complex leads to apoptosis and cell cycle arrest**. Knockdown of h*NAA10 *leads to reduced acetylation of hNaa10p subtrates [9]. As a direct or indirect effect of this, apoptosis and cell cycle arrest is observed [8]. Direct acetylation of β-catenin has been implicated in the cell cycle arrest mediated by h*NAA10 *knockdown [35]. Loss of acetylation can lead to reduced Cyclin D1 expression, and subsequent cell cycle arrest in the in the G_0_-G_1 _phase. One hypothetical mechanism is that hNatA affects cell cycle indirectly via a HAT (Histone Acetyltransferase), acting on β-catenin or directly or indirectly at the level of Cyclin D1 expression. This is more thoroughly issued in a previous publication [40]. More experimental studies are needed to determine the mechanisms through which hNatA knockdown phenotypes are formed.

A general function of the hNatA complex has not been assessed, but knockdown studies have pointed to some important cellular processes that are affected by hNatA. Figure 2 gives an overview of phenotyphes after h*NAA10 *knockdown.

Knockdown of h*NAA10 *in HepG2 cells gave suppression of genes involved in cell growth and survival (*BECN1*, antiapoptotic protein beclin, and the *TPD52 *marker of cell proliferation), and metabolism. Among the mostly upregulated genes were antiproliferative gens such as *BIK *(negative regulator of cell growth), and a group of extracellular matrix genes [37]. In addition, several genes involved in cell cycle, growth, and survival were positively regulated as a response to h*NAA10 *overexpression: *CDC2 *(cell division cycle 2), *CCNA2 *(Cyclin A2) and *BIRC5 *(apoptosis inhibitor surviving) [37].

Lim and colleagues found that h*NAA10 *RNAi in human lung cancer cells H1299 and A549 inhibited cell proliferation and arrested cells in the G_1_-phase. Cyclin D1 was also suppressed in growth-arrested cells. This was connected to repressed promoter activity of *CCND1 *gene. It was shown that binding of the β-catenin/TCF4 transcription factor complex to the CCND1 promoter was positively regulated by hNaa10p, and negatively regulated after h*NAA10 *RNAi downregulation [35]. These data suggest that hNaa10p promotes proliferation of lung cancer cells through the β-catenin pathway.

In HeLa cells, knockdown of h*NAA10*, h*NAA15 *and h*NAA16 *induced apoptosis through the caspase dependent pathway, and possibly through G_0_/G_1 _cell cycle arrest [8,12]. In HeLa S3 cells, knockdown h*NAA10 *did not lead to apoptosis, but induced G_0_/G_1 _cell cycle arrest and sensitization to daunorubicin-induced apoptosis [8]. On the other hand, it was reported that hNaa10p have a proapoptotic function in DNA-damage induced apoptosis by working as a caspase modulator. *Drosophila NAA10 *was found among genes involved in caspase-dependent cell death after prolonged doxorubicin treatment of *Drosophila *Kc cells [41]. In contrast to the results from Arnesen and colleagues, the viability of HeLa cells was not affected by h*NAA10 *RNAi treatment. Furthermore, cells turned to be less prone to apoptosis under doxorubicin treatment [41]. More experimental evidence is needed to clear up these phenotypic differences. Finding and verification of hNaa10p specific substrates related to apoptosis will also be of great value.

In conclusion, hNatA plays an important role in cell cycle and cell growth. Loss of hNatA activity leads to decreased cell viability, and in many cases, apoptosis (Figure 2). During apoptosis, both hNaa10p and hNaa15p are cleaved, presumably by caspases, resulting in decreased NatA activity [1]. Knockdown studies of h*NAA10*, h*NAA15 *and h*NAA16 *in human cells did not show compensatory expression of corresponding paralogs h*NAA11*, h*NAA15 *and h*NAA16*, thereby suggesting independent regulation of these genes [12,37].

### Expression of h*NAA10 *and h*NAA15 *is correlated with tumor development

h*NAA10 *and h*NAA15 *expression levels are higher in several types of tumors. Indeed, several studies have shown that h*NAA10 *and h*NAA15 *expression correlates with aggressiveness of tumors. This is consistent with the above-mentioned correlation between h*NAA10 *and h*NAA15 *expression and cell proliferation.

h*NAA10 *was significantly overexpressed in hepatocellular carcinomas. In the same study h*NAA10 *was identified among genes associated with dedifferentiation of hepatocellular carcinoma [42]. By immunohistochemistry, hNaa10p was shown to be overexpressed in colorectal cancers as compared to adjacent normal tissue [43]. An other study demonstrated that hNaa10p was overexpressed in colorectal cancers as well as in breast cancers [44]. The hNat10p upregulation was especially significant in cells with epithelial origin. Comparison studies on glandular carcinomas and squamous carcinomas originating from the same tissue concluded that the high positive rate of hNaa10p expression was lower in glandular carcinoma than in squamous cancer [44].

h*NAA15 *was originally identified as a gene overexpressed in gastric cancer [26] and papillary thyroid carcinomas, especially in clinically aggressive tumors with histological evidence of poorly differentiated or dedifferentiated areas [10]. hNaa15p expression levels were also higher in thyroid neoplasms (follicular adenomas and papillary thyroid carcinomas) [21]. High levels of hNaa15p protein expression were observed in neuroblastomas, with unfavourable histopathology and advanced stage. Furthermore, the expression level of hNaa15p was found to correlate with high-risk groups and poor outcome [31].

When comparing the mRNA expression of h*NAA15 *and h*NAA16 *in thyroid cell lines from normal fetal and adult thyroid cells; follicular, papillar and anaplastic thyroid carcinomas, h*NAA15 *was found to be slightly overexpressed in all thyroid cancer cell lines compared to expression in primary thyroid cell lines. No similar overexpression was observed for h*NAA16 *[12].

Taken together, the hNatA subunits seem to play important roles in cell proliferation and potentially in tumorigenesis. They may be important biomarkers for various forms for cancers. hNatA subunits can also be potential targets for cancer drug treatment, as previously summarized by Arnesen and colleagues [40]. To better understand in which processes hNatA participate, it would be of utmost importance both to establish functional links from specific hNatA substrates to cellular mechanisms, and to understand the complete cellular effect of hNatA acetylation.

## The human NatB complex

The human NatB complex (hNatB) was recently identified [3,45]. It is composed of the catalytic subunit hNaa20p (hNAT3), and the auxiliary subunit hNaa25p (hMDM20). This complex is conserved from yeast both with respect to subunit composition and substrate specificity.

Both subunits are found in the cytoplasm, where they are present both in a ribosome-bound and a non-ribosomal form. The substantial amount of hNaa20p and hNaa25p present in the non-polyribosomal fraction indicates that these proteins dynamically interact with the ribosome and/or have other functions independent of ribosome binding [3].

### Composition of the hNatB complex

#### hNaa20p

hNaa20p has a theoretical molecular mass of 20.4 kDa, and contains a conserved acetyltransferase domain. hNaa20p localizes to both cytoplasm and nucleus, and it is homologous to the yeast NatB catalytic subunit yNaa20p [3].

#### hNaa25p

hNaa25p has a theoretical molecular mass of 112.3 kDa. It has been predicted to contain two globular domains, a TPR-region, and a nuclear localization signal. Even so, hNaa25p has a cytoplasmic localization, and it has not been observed in the nucleus. The hNaa25p protein shares 20.4% and 92.9% sequence identity with its yeast and mouse homologues, respectively, indicating a high degree of evolutionary conservation within higher eukaryotes, and a moderate degree of sequence conservation from yeast to human [3].

### Substrates of hNatB

The hNatB complex was found to *in vitro *acetylate a peptide with an MDEL N-terminus [3]. This represents the N-terminus of the NF-κB subunit p65. p65 is also *in vivo *acetylated in HeLa cells [9]. Indeed, the identification of many acetylated Met-acidic N-termini in a large scale proteomics analysis [9], including the MDEL N-terminus, suggest that this activity is dependent on the hNatB complex. As the yNatB is known to acetylate methionines that are followed by an acidic residue, the acetylation of the MDEL-peptide supports a conservation of substrate specificity of NatB from yeast to man [46].

### Knockdown of hNatB induces cell cycle arrest

**Figure 3:**
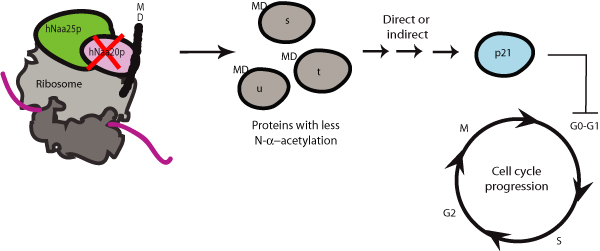
**Knockdown of h*NAA20 *of the hNatB complex leads to inhibition of growth and cell cycle arrest**. Knockdown of h*NAA20 *may lead to reduced acetylation of hNaa20p substrates. As a direct or indirect effect of this, inhibition of cell growth, and G_0_-G_1 _cell cycle arrest is observed. The finding that p21 is upregulated after h*NAA20 *knockdown suggest a mechanism where loss of hNaa20p mediated acetylation leads to p21-mediated cell cycle arrest.

Knockdown of hNatB subunits inhibits cell growth and proliferation, and disturbs cell cycle progression. Knockdown of h*NAA20 *leads to G_0_/G_1 _arrest, and an increase in the level of p21 (as summarized in Figure 3). Knockdown of h*NAA25 *leads to cell death, a decrease in G_0_/G_1 _cells, and a decrease in p21 levels. Also, a decrease in hNaa20p levels was observed after h*NAA25 *knockdown, indicating that hNaa25p is needed for hNaa20p stability [3]. This also implicates that h*NAA25 *phenotypes will include h*NAA20 *phenotypes. The change in p21 levels indicate that at least some of the effects of hNatB knockdown are mediated through p21, which is a known inhibitor of traverse through the G_1_-phase [47]. The seemingly contradictory effects of h*NAA20 *and h*NAA25 *knockdown can be the result of complex downstream mechanisms for the hNatB. Elevated levels of p21 may inhibit the induction of apoptosis while in some cases it is known that preventing induction of p21 is necessary to induce apoptosis [48]. Thus, the p21 level may explain why we detect cell death when analysing the h*NAA25 *knockdown cells, while no significant levels of apoptosis are detected for h*NAA20 *knockdown cells. The differences in phenotypes could also indicate that one or both hNatB subunits may have individual functions in addition to those of the hNaa20p-hNaa25p complex. The observation that h*NAA20 *knockdown leads to cell cycle arrest was also confirmed in other studies [45]. Further information regarding hNatB can be found elsewhere in this supplement [49].

## The human NatC complex

The human NatC complex (hNatC) was recently described. It consists of the catalytic subunit hNaa30p (hMak3), and the auxiliary subunits hNaa35p (hMak10) and hNaa38p (hMak31) [4].

hNatC is conserved from yeast to man both with respect to subunit composition, and substrate specificity, as is also the case with hNatA and hNatB. hNaa30p and hNaa35p localize almost exclusively to the cytoplasm while hNaa38p also localizes to the nucleus. In the cytoplasm, all three subunits are found both in a ribosome-bound and a non-ribosomal form. This supports a model where the hNatC complex co-translationally acetylates nascent polypeptides. As the subunits are present also in a non-ribosome-bound form, they may dynamically interact with ribosomes, and/or have functions independent of ribosomal binding as also suggested for the hNatA and hNatB subunits [4].

### Composition of the hNatC complex

#### hNaa30p

The N^α^-acetyltransferase hNaa30p (hMak3) (GeneID 122830) is a protein with theoretical molecular weight of 39.3 kDa. hNaa30p acetylates peptides with N-termini MLALI, MLGTG and MLGTE [4]. This substrate specificity is similar to that observed for yNaa30p, thus Naa30p is evolutionary conserved in eukaryotes. Also, these *in vitro *experiments showed that hNaa30p was enzymatically active without being associated with hNaa35p and hNaa38p, showing that the substrate specificity of hNatC is at least partly contained within the catalytic subunit itself. Pesaresi and co-workers demonstrated that the *A. thaliana *Naa30p (AtNat30p) alone was able to functionally replace the yeast NatC complex. Also, in contrast to AtNaa30p, knockout of AtNaa35p alone did not result in any obvious defects [50]. This indicates that Naa30p may have functions independently of the NatC complex in higher organisms than yeast.

Interestingly, hNaa30p (362 amino acids) is considerably larger than its yeast homolog (176 amino acids). This is mainly due to an additional N-terminal region of hNaa30p. Similarly, the *A. thaliana *Naa30p also contains additional residues as compared to yeast Naa30p, and AtNaa30p displayed enzymatic activity independently of AtNaa35p [50]. The function of this N-terminal domain hNaa30p domain is unclear, but it contains several potential phosphorylation sites, making it a possible region for posttranslational regulation of hNaa30p activity, as was observed for hNaa10p [16].

#### hNaa35p

hNaa35p (hMak10) is the human homologue of the yeast NatC subunit yNaa35p (yMak10p). The experimentally described h*NAA35 *differed from the predicted sequence available at Entrez in that a splice event had taken place resulting in the loss of nucleotides 1465–1704 as compared to the predicted sequence [4].

The rat *NAA35 *homologue was identified as a novel gene upregulated in the healing corneal epithelium. Its expression correlated with reepithelialization of cornea and maturation of the cornea and skin suggesting the role of this gene in epithelial development, differentiation, and wound healing [51].

#### hNaa38p

hNaa38p (hMak31, LSMD1) is the human homologue of yeast NatC subunit yNaa38p (yMak31p). hNaa38p is a member of the Sm and Sm-like proteins which associate with RNA and are involved in RNA-processing event. As hNaa38p is also found in the nucleus, it may have a nuclear role in RNA processing independent of the hNatC complex [4]. It is puzzling why an Sm-like protein is a part of the NatC complex. One could picture a model where the RNA-binding capacities of hNaa38p participate in the interaction between the NatC complex and factors in the translation process. An interesting question in such a scenario is why only the hNatC complex contains this factor (or indeed, why the three NAT complexes differ in their auxiliary subunits). Do the auxiliary subunits of the NAT complexes mediate complex-specific anchoring to the ribosome? The question of how the NATs are associated with the ribosome is exciting. Answering this will be important to fully understand how N^α^-terminal acetylation is facilitated.

### hNatC substrates

N^α^-terminal acetylation and myristoylation facilitate membrane association for several types of GTPases [52]. It was recently shown that the human Arf-like GTPase Arl8b (hArl8b) depended on its acetylated N-terminus for proper lysosomal association [53]. The N-terminal sequence of hArl8b is MLAL, matching the substrate specificity of the NatC complex from yeast: methionine followed by a hydrophobic amino acid residue [7]. Thus, hArl8b is a potential hNatC substrate. Indeed hNaa30p acetylates an MLAL N-terminus *in vitro*. Also, h*NAA30 *knockdown leads to aberrant lysosomal targeting of hArl8b [4]. This points to a direct link between hNatC mediated N^α^-terminal acetylation and proper functional localization of hArl8b. Another human protein of which the acetylation of the N-terminus by hNatC may be functionally important is the human GTPase Arf-related protein 1 (ARFRP1). ARFRP1 depends on its N-terminal acetylation for proper Golgi association and its N-terminus matches NatC substrate requirements [54].

Another important potential hNatC substrate is the protein kinase mammalian Target Of Rapamycin (mTOR). Wenzlau and colleagues found that knockdown of z*NAA35 *leads to loss of phosphorylation of downstream mTOR substrates. Pharmacological inhibition of TOR with rapamycin showed similar phenotypes as z*NAA35 *mutants with respect to growth and vessel defects [55], thus defects in the TOR pathway may partly explain the *NAA35 *knockout phenotypes.

### Expression of the hNatC subunits

EST data from UniGene Cluster indicate that h*NAA30 *(Hs. 165465), h*NAA35 *(Hs. 436098), and h*NAA38 *(Hs. 565094) are ubiquitously expressed in epithelial tissue, loose and dense connective tissue, and in muscle and nervous tissues. h*NAA30*, h*NAA35 *and h*NAA38 *are found co-expressed in tissues, suggesting that expression is due to hNatC function. An exception is the pituitary gland, where h*NAA35 *and h*NAA38*, but not h*NAA30*, are significantly expressed. One may speculate if hNaa35p and hNaa38p have functions independent of hNaa30p in the pituitary gland. Gene expression of h*NAA30 *and h*NAA35 *at mRNA level measured by RT-qPCR is in accordance with EST data, confirming ubiquitous expression of these genes in all analyzed human cancer cell lines (Gromyko D. *et al*., unpublished data).

### Knockdown of hNatC induces apoptosis in human cell lines

Knockdown of each of the hNatC subunits leads to similar phenotypes in HeLa cells: reduced cell proliferation and apoptosis [4]. This suggests that the observed knockdown phenotypes indeed are due to the loss of hNatC activity, and that all three subunits are needed for hNatC activity. A stronger phenotype was observed in cells with downregulated catalytic subunit hNaa30p, as compared to knockdown of auxiliary subunits hNaa35p and hNaa38p. Taken together, these phenotypes suggest that hNatC is required for normal cell growth and survival.

**Figure 4:**
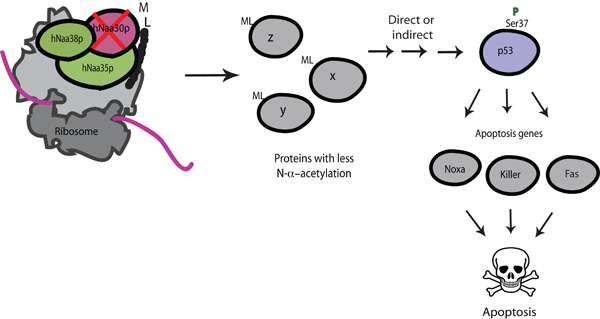
**Knockdown of h*NAA30 *of the hNatC complex leads to p53-dependent apoptosis**. Knockdown of h*NAA30 *may lead to the reduced acetylation of hNaa30p substrates. As a direct or indirect effect of this, p53 is stabilized and phosphorylated on Serine 37, and subsequently, transcription of p53 downstream proapoptotic genes *NOXA, KILLER *and *FAS *is activated, resulting in apoptosis.

Furthermore, downregulation of hNatC subunits independently of one another in colon carcinoma cell lines HCT116 (*TP53+/+ *and *TP53-/-*) demonstrated association between wild type* TP53 *and apoptosis: the observed apoptotic phenotype was dependent on a functional *TP53 *[4]. In addition, our findings indicated an activation of *TP53 *in cells with downregulated levels of hNaa30p. After h*NAA30 *knockdown we observed an increase in p53 protein level, an increase in p53 Serine 37 phosphorylation, and an increase in the expression of proapoptotic p53-downstream genes *KILLER, NOXA *and *FAS*. It is unclear how lack of hNatC-mediated acetylation causes activation of *TP53*, and thereby expression of downstream proapoptotic genes. One scenario may be that hNatC mediated acetylation is needed for normal function of factors upstream of p53. A summary of hNaa30p knockdown phenotypes is given in Figure 4.

In zebrafish, the knockdown of the h*NAA35 *homologue Embryonic Growth-Assiciated Factor (*EGAP*, z*NAA35*) leads to embryonic lethality due to decreased cell proliferation, increased apoptosis, and poor blood vessel development [55]. These findings emphasize the importance of NatC for normal cell function and development in higher eukaryotes. Further studies are needed to understand the function of hNatC, and the mechanisms through which hNatC knockdown phenotypes are mediated.

## The human NatD and NatE complexes

In yeast, two more NATs, NatD and NatE, have been described.

### NatD

NatD is the N^α^-acetyltransferase Naa40p (Nat4p). It was recently described to acetylate the Ser- N-termini of histones H2A and H4 in yeast [56]. No auxiliary subunits have been presented. To our knowledge, no studies have so far characterized the hNaa40p/hNatD activity in human cells. However, based on homology predictions, there exists a human homologue to the *NAA40 *gene [56].

### hNatE

NatE is the designation for a complex consisting of the putative N^α^-acetyltransferase Naa50p (Nat5p), and Naa10p and Naa15p of the NatA complex. hNaa50p is the human homologue of the yeast Naa50p (Nat5p), and the fruitfly San protein [24,57]. hNaa50p is predicted to be a N^α^-acetyltransferase, but this has not been verified experimentally. Naa50p is physically associated with Naa10p and Naa15p in several species including humans [5,24,57], but knockdown of Naa50p does not affect NatA type acetylations in lower eukaryotes. Thus, it is hypothesized that Naa50p has functions distinct from those of the NatA complex. However, the presence of Naa10p and Naa15p may be obligatory for the stability of Naa50p in yeast and humans [5,6,24]. In humans, knockdown of h*NAA50 *resulted in less severe and more distinct phenotypes as compared to hNatA (h*NAA10*-h*NAA15*) knockdown, further suggesting that hNaa50p functions separately from the hNatA complex [6]. More specifically, studies from both fruitflies and humans suggest a role for Naa50p in centromeric cohesion [6,57]. Proper sister chromatid cohesion depends on the acetyltransferase activity of hNaa50p, but this activity has so far not been classified as a HAT or NAT activity [6]. Recently, Drosophila Naa50p was also shown to have a more general role in chromosome resolution [58]. So far, no substrate of Naa50p from any species has been identified, thus the final confirmation that this is indeed a NAT awaits further investigations.

## Summary and conclusion

The field of human NATs is still in its infant steps. During the last five years the major NAT complexes hNatA, hNatB, and hNatC have been described. This represents a significant leap forward in the knowledge of N^α^-terminal acetylation. As to this date, the minor hNatD and hNatE complexes remain unstudied. Bringing hNatD and hNatE along will be important, especially hNatE (hNaa50p), since this NAT type is completely uncharacterized for all species with respect to substrate specificity. With the NatA to NatE complexes characterized, it is likely that all NAT types in lower eukaryotes are described, since all N-terminally acetylated proteins are accounted for, and because there are no additional genes in the *S. cerevisiae *genome that are likely to encode additional NATs. However, for humans and other higher eukaryotes it is not unlikely that additional NATs exist. This is based on the fact that more substrates are N-terminally acetylated in humans as compared to yeast [9]. For example, Met-Lys N-termini are unacetylated in yeast while a portion of these N-termini is acetylated in human cells. Another area of uncertainty in humans is the N-terminal acetylation of actins. These acidic N-termini are acetylated, most likely post-translationally, by a so far unknown NAT. Finally, not all N-termini of Met-Ser, Met-Ala- types have their Met- cleaved by MetAPs, and many of these N-termini are acetylated in humans. These classes of substrates may indeed be N-terminally acetylated by hNatA-hNatE activities. They may also very well be acetylated by novel human NATs yet to be described, like the Camello class of proteins present in higher eukaryotes, which displays sequence similarity to the NATs [7].

Even though knockdown phenotypes of all major human NATs suggest important roles for these complexes, functional links between specific substrates and the phenotypes have not been established. Clearly, this represents challenge of great importance. Recent works estimate that 80% of all soluble human proteins are N-terminally acetylated. The human NATs display severe knockdown phenotypes, and have several potential links to disease. Thus, this modification and these enzymes clearly deserve significant attention in the future.

## Competing interests

The authors declare that they have no competing interests.

## Authors' contributions

KKS and DG wrote the manuscript draft. All authors read and corrected the manuscript, and approved the final version.
